# Health From the Perspective of Adolescents With Intellectual Disabilities—Report From Poland

**DOI:** 10.1111/jar.70119

**Published:** 2025-09-08

**Authors:** Beata Gumienny, Aneta Lew‐Koralewicz

**Affiliations:** ^1^ University of Rzeszów Institute of Pedagogy Rzeszów Poland

**Keywords:** adolescents, health, intellectual disability, interpretative phenomenological analysis

## Abstract

**Background:**

Health awareness is an important factor in preventive health and healthy lifestyles of children and adolescents with an intellectual disability. The research objective is therefore to explore the perspective of people with intellectual disability regarding their health‐related experiences and the meanings they assign to health.

**Methods:**

Using interpretative phenomenological analysis (IPA) as a methodological approach, semi‐structured interviews were conducted with 14 students between the ages of 13 and 19.

**Results:**

Analysis identified four themes: understanding of health, perceptions of a healthy lifestyle, identifying health‐at‐risk situations, and experiencing illness. For them, health means feeling good, the lack of illness, and a healthy lifestyle. Participants can identify various health‐threatening situations.

**Conclusions:**

It is necessary to strengthen health education and health awareness in the population of people with intellectual disability, so as to enable them to increase the scope of their autonomy and self‐determination with regard to protecting their own health.

**Summary**

- People with intellectual disability perceive health as a state of well‐being, the absence of illness, as well as the ability to participate in daily activities.
- Adolescents with intellectual disabilities recognise the importance of a healthy lifestyle.
- Participants know the symptoms of the most common diseases and how to deal with them, but rely on the support of relatives.
- It is crucial to increase the level of knowledge and develop correct health behaviours so as to increase the autonomy of participants.

## 1 Introduction

Health is of particular value in any individual's life. People with intellectual disability require additional support and attention in the area of healthcare due to their specific functioning resulting from their clinical symptoms. Intellectual development disorder is characterised by significantly below‐average intellectual functioning and adaptive behaviour that is approximately ≥ 2 standard deviations below average. These people constitute a significant group, as the overall prevalence of intellectual disability is about 1.93%, although this is known to vary according to age group and income status in a given country (Mizen 2024). As research shows, some health problems are more common in people with intellectual disability than in the general population. This often includes gastrointestinal problems, as well as excess weight and obesity and the resulting health consequences (Haveman et al. 2010; McGuire et al. 2007; Young‐Southward et al. 2017). These problems can be the result of both preferred lifestyles and problems in accessing health education, as well as the lack of appropriate prevention and health care.

World Health Organization defines health as a state of complete physical, mental and social well‐being and not merely the absence of disease or infirmity (WHO). Having better health is an important factor in generating a higher feeling of well‐being (Tilley et al. 2020). It is necessary to develop healthcare practices and behaviours that improve the quality of services, as well as the well‐being and health of people with intellectual disability (Bacherini et al. 2024). The right to healthcare, and free healthcare for people with intellectual disability, is guaranteed by Article 25 of the *Convention on the Rights of Persons with Disabilities*. Ensuring good quality mental and physical healthcare is one of the priorities of the *European Declaration on the Health of Children and Young People with Intellectual Disabilities and their Families: Better Health—Better Lives* approved by the WHO (2010a) and WHO (2010b).

In Polish educational law, there are provisions for the education of students with mild intellectual disability (general education regulations) and for students with moderate and severe intellectual disability (separate regulations), which stipulate that physical education is compulsory and plays a leading role in health education. In addition, the education system must provide classes on health, rational nutrition, physical activity, safety and hygiene (Dz 2017 poz. 356).

Despite good legislation aimed at equal access to healthcare, people with intellectual disability experience a variety of barriers to accessing the healthcare system, which adversely affects their quality of life and the well‐being of their families (Gajdzica et al. 2023). People with disabilities experience unfair treatment, discrimination, lack of reasonable treatment from health services, as well as communication barriers and problems accessing services (Ali et al. 2013; Cytowska and Zierkiewicz 2020; Krahn and Fox 2014; Ziviani et al. 2004). Many factors contribute to reducing the quality of healthcare interventions for people with intellectual disability. These relate to the attitudes of health professionals, the limited availability of health information, the level of involvement of support persons, system limitations, person‐centred informed consent, and effective communication between health care professionals and patients (Dunn et al. 2023).

Health awareness plays an important role in interactions, from prevention and health promotion to coping with illness. Improving knowledge about health is important from the perspective of developing the autonomy and independence of people with intellectual disability and preparing them to live independently and make their own decisions about their health behaviours. According to researchers, a prerequisite for people with intellectual disability to be healthy in adulthood is the ability to live life according to their own preferences and make independent choices while receiving adequate support (Johansson et al. 2017).

Health awareness allows for the development of health behaviours, which have been defined as “those personal attributes such as beliefs, expectations, motives, values, perceptions, and other cognitive elements; personality characteristics, including affective and emotional states and traits; and overt behaviour patterns, actions, and habits that relate to health maintenance, to health restoration, and to health improvement” (Gochman 1982, as cited in Glanz et al. 2008, 12).

The health behaviour of people with intellectual disability is determined by many factors (Mach 2017). Due to challenges with cognitive and social functioning, people with intellectual disability may experience difficulties in developing their own health behaviours. An important aspect of working with adolescents with intellectual disability is the formation of their health behaviours in the area of health prevention, behaviour related to the identification of diseases and treatment options, as well as behaviour in the role of the patient (see Glanz et al. 2008).

Effective interventions in the process of healthcare and promotion require taking into account the autonomy of people with intellectual disability and their need for self‐determination. People with intellectual disability desire self‐determination regarding their medical health care trajectory (Pouls et al. 2024). Increasing the autonomy of people with intellectual disability therefore becomes an important aspect of interventions in the area of health behaviour development. A prerequisite for developing health behaviours and taking care of one's own health is to be able to live life according to one's preferences and make independent choices while receiving adequate support (Johansson et al. 2017; Wrzesińska et al. 2024). Research shows that people with intellectual disability want to be included in primary healthcare through empowerment, appropriate communication, relational ties, reasonable adaptations and better access to medical support (Downs et al. 2024; Gregson et al. 2024); however, there are significant limitations in this regard (Wullink et al. 2009).

Health literacy skills enable people with intellectual disability to make rational decisions about prevention and healthcare, and to access the services of the healthcare system. Understanding how they perceive health issues and the meanings they assign them is crucial for planning educational and support activities.

## 2 Material and Methods

Qualitative methods are playing an increasingly significant role in the study of intellectual disability and, in particular, in increasing our knowledge of as yet unknown areas of functioning. This approach needs to be expanded, as currently the views of people with intellectual disability have not been completely explored (Diaz et al. 2024).

### 2.1 Study Design and Participants

Interpretative Phenomenological Analysis is a method designed to understand people's life experience and how they make sense of it in the context of their personal and social worlds (Smith et al. 2009; Smith and Nizza 2022, 3). The theoretical foundations of IPA are rooted in phenomenology, hermeneutics, and idiography. Phenomenology is a philosophical method for studying human experience, emphasising the need to explore it as it occurs and on its own terms, rather than through predetermined theoretical frameworks. The emphasis is placed on investigating lived experience through conscious awareness and reflection. Hermeneutics is the theory of interpretation. The analytical process in IPA is commonly referred to as a double hermeneutic or dual interpretation process. First, participants interpret their world, and second, the researcher seeks to decode that interpretation to understand the meaning participants have constructed. Idiography is an approach in research that focuses on the detailed and in‐depth study of individual cases or specific phenomena. There is an interest in understanding the experience of particular people in particular circumstances and a belief that this is best achieved by focusing on single cases to be analysed individually before possibly making comparisons between cases (Smith et al. 2009). When implemented with methodological rigour, IPA can be successfully applied to research on intellectual disabilities. IPA provides an opportunity to listen to the voice of people with intellectual disability, to learn about their preferences and needs, as well as the meanings they give to their experiences (Corby et al. 2015; Rose et al. 2019).

The purpose of the research is to explore the issue of health in the perception of people with intellectual disability. The value of exploring the life experiences of young people with intellectual disabilities, in the context of health, lies in particularly understanding how they make sense of and interpret health‐related experiences. Gaining insight into their individual perspectives on health behaviours could aid persons who work with children with intellectual disabilities in formulating preventive and educational strategies aimed at developing pro‐health knowledge and skills. This in turn promotes self‐determination, person‐centred planning, and an enhanced quality of life for individuals with intellectual disabilities.

The qualitative study conducted seeks to address the following research questions: What meanings do students with intellectual disabilities attribute to health, and what are their experiences related to it? How do they perceive situations that pose a threat to their safety and health? A semi‐structured, problem‐focused interview was used.

The interview guide included topics such as: What does health mean to you? How do you define healthy living? How do you maintain your health? What factors can jeopardise your health? How do you handle situations involving illness? The questions were general in nature, but throughout the interview, additional supportive and specific questions were asked based on the individual needs of the participants.

The study involved 11 people with moderate and 3 with mild intellectual disability. The diagnosis of intellectual disability was confirmed at a psychological‐educational counselling centre in early childhood. As suggested by Smith et al. (Smith et al. 2009), the sample size should be between 6 and 10 participants; however, if the quality or depth of the data is more variable, then the group can be larger. This is applicable in the case of this research, due to the varying levels of linguistic and communicative competence. Specific information about the participants is presented in Table 1.

**TABLE 1:** Participants' characteristics.

Participant	Gender	Age	Level of intellectual disability	Educational establishment
K1	Female	17	Moderate	Special vocational training school
K2	Female	19	Moderate	Special vocational training school
K3	Female	19	Moderate	Special vocational training school
K4	Female	15	Moderate	Special school
M1	Male	17	Moderate	Special vocational training school
M2	Male	18	Moderate	Special vocational training school
M3	Male	18	Moderate	Special vocational training school
M4	Male	17	Moderate	Special vocational training school
M5	Male	13	Mild	Integrated school
M6	Male	18	Moderate	Special vocational training school
M7	Male	17	Moderate	Special vocational training school
M8	Male	17	Moderate	Special vocational training school
M9	Male	13	Mild	Integrated school
M10	Male	14	Mild	Special school

### 2.2 Procedure

Approval from the Bioethics Committee of the University of Rzeszow was obtained prior to the study. Nonprobability sampling techniques were used due to the criterion of certified intellectual disability and the level of communicative competence. Individuals were recruited through educational and therapeutic institutions designed for people with disabilities. Permission was obtained from the directors of the institutions to conduct the study, and informed consents from the research participants were collected by six teachers or therapists who qualified the students for the study. Four were specialists from the special vocational training school, one person worked in a special school and another in an integrated school. The recruitment for the study was managed by teachers and therapists who used their own discretion in identifying suitable participants for the study. Sixteen students were invited to participate, but one was unable to due to a long‐term illness, and another chose to withdraw from the study without providing a reason. In the case of underage students, parental consent was also obtained for the study. The informed consent form for the research participants was designed with easy‐to‐read rules. Before signing the informed consent, the researchers made sure that the participants understood the purpose of the study and how the research data would be used, as well as consented to the recording of the interview. They were assured that they could opt out of the study at any stage. Interviews were conducted at the school or at the therapeutic centre by prior arrangement with the management and the participant. The research took place in individual therapy rooms within the participants' educational institutions, ensuring a safe and familiar environment. Each room was equipped with a desk, comfortable chairs, and natural lighting. Participants had the freedom to choose where they wished to sit, and every effort was made to create a secure and intimate atmosphere. The study was carried out by researchers who are involved in both science and clinical and educational practice. Both have experience working in practice with people with intellectual disabilities. The researchers established a good rapport with the participants, and they were recruited by the directors of the institutions. The interviews lasted between 40 min and 75 min, depending on the participants' involvement. Interviews with people with intellectual disability most often require personalised support from the researcher. Support consisted of repeating or clarifying interview questions several times, detailing questions and paraphrasing participants' statements to ensure understanding of the meaning and context of experiences. The interviews were recorded, then copied onto a hard drive and transcribed.

### 2.3 Data Analysis

The qualitative interview data was transcribed and then subjected to IPA using MAXQDA software (version 22.8.0, VERBI GmbH, Berlin, Germany). The analysis was carried out using the suggestions developed by Smith et al. and consisted of the following steps: reading and exploratory notes, formulating experiential statements, finding connections and clustering experiential statements, compiling a table of personal experiential themes (Smith and Nizza 2022). The research questions guided the data collection strategy and identified the main themes. Data were analysed inductively to explore the meanings participants attached to their experiences. An interpretive approach enabled conclusions to be drawn from the analysis of participants' statements. The initial step in the phenomenological‐interpretive analysis involved reading the interview transcripts and listening to the recordings. Through repeated readings and careful analysis, initial meaning units were identified. In the subsequent phase, specific text passages from interview transcripts were identified and related to each of the meaning units highlighted previously. Connections between each of the meaning units were investigated and categories were grouped and refined into four main themes and emerging sub‐themes from the data. These refined codes are presented in the study's findings. Throughout the various stages of the Interpretative Phenomenological Analysis (IPA), the researchers collaborated closely, engaging in thoughtful discussions on the methodology, the data, and its analysis.

## 3 Results

The problem of health belongs to areas related to the quality of life. The meanings attributed to health and how it is felt and experienced stem from subjective understanding of this phenomenon. Analysis of the collected material demonstrated what meanings students with intellectual disability attributed to health, how they interpreted their well‐being, and how they experience situations that put their safety and health at risk. The IPA process identified four main themes, as presented in Table 2.

**TABLE 2:** Superordinate and constituent themes.

**Understanding of health**
Well‐being Absence of illness Experiencing daily routine Seeing oneself through the lens of relatives
**Perceptions of a healthy lifestyle**
Physical activity, sports Healthy food Unhealthy foods Appropriate clothing
**Identifying health‐at‐risk situations**
Accidents Drugs and toxic substances Physical and psychological aggression Abuse of modern technologies
**Experiencing illness**
Familiarity with the symptoms of the disease Dealing with illness Responding to a health emergency

### 3.1 Understanding of Health

The first theme that emerged from the research is the way in which participants with intellectual disability understand health, and the meaning they give to it. In interpreting the concept of health, participants point to its positive aspects, involving well‐being, recognising that health reflected by **well‐being** is variable and unstable: “That it feels good” (*M4*), “Health, feeling better, is either worse or better” (*M3*). The need to take medication to maintain health and well‐being was also identified: “Health … if I feel good, when I take medication for my health” (*M2*) [medication taken as standard for epilepsy, for example; author's note].

Health is understood by respondents as both a physical and emotional experience, perceived directly and associated with the “here and now”, yet also recognised as a state that is subject to change. Some respondents emphasise that health is not so much a natural condition as one that is sustained through the use of medication. This perspective reflects beliefs about the dependence of health status on medical care, which may be rooted in the respondents' personal experiences.

In the statements, there is a tendency to define health through negative aspects by **denying symptoms of illness** and experiencing pain, thus emphasising well‐being. Participants also mention specific symptoms of illnesses, the appearance of which would indicate damage to health, that is, fever, sinusitis, runny nose, cough, sore throat, or muscle pain. “Health means someone is healthy, that nothing hurts him, maybe he feels good” (*M6*), “To not get sick” (*K1*), A significant health perception perspective was also identified, capturing the aspect of valuing health and its importance. The participant emphasises how important health is to her. The statement is dominated by respect for health, expressed in the phenomenon of being appreciated. *“I feel good all the time, I appreciate my health well”* (*…*) (*K3*).

Many statements define health through the negation of disease symptoms. This interpretation suggests that, for participants, health exists primarily in opposition to illness, rather than as a positive state in its own right. The mention of specific symptoms indicates that health is understood in predominantly physical terms. Health is thus anchored in corporeality—whether something hurts or is physically felt determines how health is assessed. The absence of references to mental or social aspects of health among participants may point to a limited understanding of health or to particular cognitive patterns in how they perceive it.

**Experiencing daily routines** is another way of understanding health, reflected in the statement “Life, just life” (*K3*). In a general sense, according to the participants, health refers to experiencing daily activities that signify a certain sequence of natural events, as confirmed by the following statement: “I am very healthy and I lie in bed and sleep and get up” (*M1*). It is not only ordinary daily life that constitutes the essence of life, but also the possibility of pursuing passions and dreams, as the young woman says: “A healthy life means that I am in life, that I was born, that everything is fine and I am doing everything, that I am fulfilling my dreams” (*K2*).

These accounts suggest that health may be understood as a continuity of everyday life—not as something extraordinary, but as the ability to engage in familiar, simple activities. Sleep, getting out of bed, and other routine actions become indicators of health for the participants. Health is thus associated with normality, predictability, and stability. This perspective may reflect a need for structure and routine, which can be particularly significant for individuals with intellectual disabilities. In this context, health emerges as a condition for personhood and a meaningful life, rather than merely the absence of illness.

Participants also equated health with **perceiving health through the prism of relatives**. Young people with intellectual disability perceive themselves through the prism of the opinions of significant others. Parents, especially the mother, play a unique role in this process. When conducting interviews, some participants usually began their statements with the phrase: “my mother said…”, for example, “My mother said that I am very healthy and I have a lot of strength and I read different books to myself, I write and listen to myself (…), I watch movies” (*M1*). What emerges in the statement is the essence of verbal communications formulated by mothers, who play an extremely important role in the life of a person with intellectual disabilities. The mother's opinion, her perspective of the child's perception and her explanation of the world, become an important factor in the understanding of health by people with intellectual disability.

From this perspective, health is not merely an internally perceived state, but its understanding is shaped through relationships with others. This reveals the social construction of health identity, in which a person derives a sense of health from input provided by significant others.

Four participants had difficulty defining health issues, indicating difficulties in verbalising their own meanings assigned to health.

Participants appreciate the value of health and perceive it through the prism of their experiences, giving meaning to health in positive and negative aspects. Positive meanings are associated with well‐being, which enables people to fulfil social roles and pursue their passions and dreams. For some participants, experiencing health is equated with the absence of illness.

### 3.2 Perceptions of a Healthy Lifestyle

Among the ways to identify a healthy lifestyle is to view it through the prism of **sports and physical activity**. Participants point out the importance of physical activity, but also the need for exercise moderation, which is expressed in the statements: “Healthy lifestyle, healthy workout, healthy play” (*M7*). “You can't push yourself so much in the gym, because then, I don't know, you can break your arm, or your leg” (*M10*). Among the forms of sports activity, participants listed: soccer, volleyball, basketball, table tennis, badminton, skating, cycling, running, exercising and swimming. The motives behind the physical activities undertaken are: keeping fit, losing weight, maintaining a healthy body weight, wanting to be fit and “muscular,” and being outdoors and “burning fat.” Although they are aware of the benefits of physical activity, some of them admit that they do not systematically practice any sports, and their physical activity is limited to physical education classes.

A healthy lifestyle is understood by some participants as both a means of exerting control over the body and a source of pleasure, as well as a way of connecting with the environment. The emphasis on physical activity may reflect the cultural significance attributed to bodily appearance and physical fitness. Participants also value the sensory and relaxing aspects of movement. In this context, a healthy lifestyle emerges as a delicate balance that requires rationality and moderation. The participants' accounts reveal a notable discrepancy between intention and practice, which may lead to an internal conflict: “I want to be active, but I cannot fully realise this aspiration”.

Some adolescents equate health with **healthy food**: “It is a healthy life that we eat healthily” (*M5*). “Health means that I eat healthily and watch my diet” (*K2*). Analysis of the data shows that the individual experiences of young people with intellectual disability regarding healthy food are varied, as only some of the participants have knowledge of the subject, mentioning specific fruit, vegetables and dishes. In addition to eating fruit and vegetables, adolescents also see the need to hydrate the body and enrich the diet with juices: “Drink a lot of water: (K3), *“To hydrate myself”*(*M4*). Participants refer to the need to reduce foods with a high sugar content, postulating that *”Eat less sweet, eat vegetables, fruit*“(K1), consume products containing vitamins: ”(*M3*), and note the need for moderation, limiting the amount of food: “I still just eat less, but I sometimes eat more, but I don't eat so constantly that I eat everything” (*M10*). An element of healthy eating is regularity in eating, especially breakfast, as one student mentions: “I have to eat, my dad will make scrambled eggs for breakfast, because I go to school, because I have to eat early” (*M1*). Awareness and the need for proper nutrition is important for young people, and the effect of taking care of rational nutrition is to keep the body healthy and immune, as reflected in the following statements: “not to get sick” (*K1*), “to be healthy” (*M3*), “not to get sick again” (*M4*).

Nutrition is not merely a biological necessity, but a conscious behaviour that reflects self‐care. Participants' knowledge of healthy eating varies, and their experience of it is shaped by environmental factors, making it neither self‐evident nor uniform. Health is perceived more as a set of recommendations than as an integrated lifestyle. Participants associate health with attention to food quality, which may stem from internal beliefs or external influences. Their statements indicate ambivalence and difficulty in maintaining control of a balanced diet, which is a real challenge in forming habits.

#### 3.2.1 Unhealthy Foods

Most of the participants identified unhealthy foods, among which they mentioned fatty products and those with high sugar and salt content, for example, fast food, energy drinks, chips, fries, doughnuts. Eating is associated with experiencing pleasure (*“delicious” ‐ M7*; “a person likes to eat” *‐ M8*), as mentioned by the study participants, who were aware that not every product has a beneficial effect on their health. However, it is usually taste that is the determining factor in choosing a product, or the desire to eat it and experience sensory pleasure. Despite their knowledge of unhealthy foods, participants have a varied diet that includes unhealthy products, as illustrated by the following examples: “Well, and as I remember I like to eat French fries with salt,” *And are chips healthy or not?* [asks the researcher] “Not really, but a person likes to eat” (*M8*) “And I sometimes eat doughnuts too, but not like this…, I sometimes buy them, but not every day” (*M10*).

Participants' accounts demonstrate an awareness of the health consequences associated with the consumption of unhealthy foods, yet they also expose a discrepancy between nutritional knowledge and everyday dietary choices. The analysis highlights an internal tension between the ability to distinguish between healthy and unhealthy products and the simultaneous drive for sensory pleasure and the reinforcement of established eating habits.

An important part of a healthy lifestyle is **appropriate clothing**. A factor that determines health is the selection of clothing appropriate to the season and weather conditions: “It is necessary to dress warmly, scarf, jacket, trousers and of course shoes” (*M10*), “To keep us warm, scarf, jacket, gloves, hat” (*M6*). The participants' experience shows that proper clothing is important because it contributes to maintaining health.

Respondents emphasise the practical and concrete dimension of caring for health, where clothing becomes a means of protecting the body. The experience of health is therefore not only related to nutrition but also involves caring for the body exposed to environmental influences.

In the participants' experience, a healthy lifestyle refers to a healthy diet and health care, to which they give significant importance. The knowledge they have on this subject, however, is not always reflected in their health‐promoting actions and behaviours.

### 3.3 Identifying Health‐At‐Risk Situations

Participants have knowledge of events that endanger human health and life, they mention accidents (collisions, crashes) as one of the first hazardous situations. They give numerous examples, describing dangerous incidents involving cars, trains, passengers and pedestrians: “Accidents endanger health, it will land you in hospital” (*M9*), “Accident and when someone is in a serious condition” (*M2*), “Dangerous situations on the roads” (*M7*). There were also contexts related to accidents during household or construction work such as: “mowing the grass” (*M7*), events occurring at home: “When I break a plate” (*K4*), *“fires”* (*K3*), or *“explosions”* (*M4*). Two participants pointed out that one should be *careful* “to watch out for cuts” (*M1*).

There is an awareness of everyday risks that may arise even in seemingly safe environments. Participants note that not only major disasters but also minor incidents can lead to injury. This reflects a functional understanding of health, recognising that it can be threatened in ordinary, everyday situations.

Among health‐threatening situations, participants also pointed to stimulants and toxic substances: cigarettes, alcohol, drugs, legal highs and energy drinks. One of the adolescents says the following about this: “Because then, if you take the wrong medicine, but I know that you must not take medicine from others, because they can be drugs. They are very harmful from strangers” (*M10*).

Another health‐threatening event identified by participants involves **physical and emotional aggression**: “It is also necessary to defend yourself, because someone may attack you” (*M3*), “That someone hits someone, or says something unkind” (*M4*). Health can be threatened by the use of physical force by others, which involves not only feeling pain, but also loss of health.

**The abuse of modern technology** was also identified as a health risk. Participants indicated that it could lead to: “deterioration of eyesight, addiction and electrocution” (*M10*), and that the dangers come from excessive playing on the phone, using instant messaging such as Messenger, WhatsApp and other apps, and general use of electronic equipment for example, tablet, computer. In the experience of young people with NI, the use of modern technology is an integral part of life in modern society, but it comes with risks.

Participants in the study demonstrate awareness of various types of health risks—from traffic accidents and violence to psychoactive substances and the impact of technology. Their perceptions are realistic and frequently grounded in everyday experiences. The individuals with intellectual disabilities surveyed are able to recognise, verbalise, and analyse health risk situations, which provide an important foundation for further educational and preventative activities.

It should be noted that a certain schematism and difficulty in interpreting phenomena related to health risks were noted in the statements. Some participants had considerable challenges in identifying health‐threatening situations.

### 3.4 Experiencing Illness

The participants are **familiar with the symptoms of disease**, listing: coughing, abdominal pain, nausea, vomiting, diarrhoea, fever, sweating, malaise, weakness, excessive fatigue, sneezing, pale and flushed skin, headaches, sore throat, legs, arms and back, trembling hands, red eyes. Illness is the experience of unpleasant conditions, associated with functional weakness of the body, malaise, increased body temperature and adverse symptoms, for example, in the respiratory or digestive systems. Some students also pointed out the causes of illness, for example, “virus, coronavirus, poisoning, germs”. Some pointed out “that you could infect someone if there was a virus” (*M7*), “because someone infects us (…), it passes on to us and we get sick” (*M10*). These statements stem from the experience of the Covid‐19 pandemic, which in a way sensitised students to the danger that a viral disease can bring.

**Dealing with illness** is reflected in one participant's indication: “Well, then medication must be taken” (*K1*). Participants are aware of the need to take medicine, of medical consultation, the course of examinations and following the doctor's instructions. Almost all of them say that if they have a developing condition, it is necessary to go to the doctor, and sometimes to hospital, for example, “Illness, then it is necessary to go to the hospital and doctors help” (*M2*). When sudden and serious health problems occur, participants are aware of the need to call the emergency medical services, for example, “when there is something more serious, you need to call the emergency medical services” (*M8*), “to the emergency medical services “(*M9*). For most people with intellectual disabilities, dealing with illness involves going to the family doctor, then to the pharmacy with a prescription and afterwards taking medicine, for example, “Medicine has to be taken. We go to the doctor and then the doctor writes us a prescription and the medicine has to be taken” (*K1*).

Participants are able to recognise and name many physical symptoms of illness, which stems from their direct experience of being unwell. Alongside these symptoms, they draw attention to the emotional aspects and the psychological dimension of illness. They also emphasise the importance of an organised support system, in which the patient does not act independently but is cared for by others—family and medical professionals.

Participants include both pharmacological treatment methods and natural methods. They take both medications that are usually given to them by their mother, mainly pills and syrups (*M4*), and indicate the need to “drink chamomile and linden herbs” (*M10*) and “tea with lemon and honey” (*M2, M10, K2, K3*). Some note that illness is associated with slowing down the pace of life and changing their daily activities, specifically “not going to school”, “sitting at home” and “lying in bed” and “watching TV and movies” (*M1, M2, M3, M4, M6, M9, K3*). Some note that experiencing various illnesses, including a weakened body, also arouses strong emotions and stress: “I feel sick then, start to get weak and sweat terribly” (*M8*), “Mum said there is an illness. I'm very scared” (*M1*), “Then I'm sad, until it makes me cry, I feel bad, I feel like resting. Then I tell my mother, my mother supports me and helps me, she always brings me tea delicious with honey and lemon, and this then helps me to de‐stress” (*K2*). Death as a consequence of illness, for example, especially from cancer and a heart attack, was reflected in the young people's statements: “They can get cancer and die” (*M9*), “When the heart stops and then you can die” (*M2*), as well as pandemics: “They were just dying. They had to go to hospital and sat in the hospital for a long time until they died. (…) half a million people died, because it's not like coronavirus and I'm going to get cured of it quickly” (*M10*).

Participants distinguish between pharmaceutical and natural remedies, indicating experience with different treatments. For many of them, illness means a departure from their daily routine, which also implies a social dimension related to isolation, changes in roles, and a different daily rhythm. Illness evokes strong emotions such as fear, sadness, and stress. All this shows that participants are aware of the serious health effects despite a simplistic understanding of biological processes. Emotional support, especially from family, plays an important role in the recovery process.

The next subcategory is responding to health emergencies. Almost all participants demonstrated some level of knowledge in dealing with life‐threatening situations. Most of the young people, using their own experience, described specific actions in the case of burns and cuts, most often demonstrating the need to disinfect (sanitise) the wound and secure it with a plaster, dressing or bandage to heal the skin and stop the blood, such as: “When I get a burn, well I pour water on it and use a plaster to seal it” (*K1*), “I stick a plaster” (*K4, M4*), “I take a plaster and pour hydrogen peroxide on it so that there is no infection, put ointment, an antibiotic, seal it and until it heals”; “Once I got burned with an iron and quickly foam gave” (*K3*), “Ice must be put on and kept for a long time” (*M6*). People with intellectual disability have knowledge of how to deal with injuries and skin damage, as well as how to call for help in situations of more serious danger. Participants indicated the need to call an ambulance.

The participants demonstrated knowledge of emergency first aid, particularly in relation to burns and cuts. Their statements reflect personal experience and an understanding of basic hygiene and protective measures. Their accounts indicate both self‐reliance and an awareness of the need to seek assistance. However, it may be assumed that the skills described are primarily declarative.

The knowledge they have and the meanings they attribute to experiencing illness stem from their own and their family's practical experiences. Participants interpret situations based on their personal life experiences, but they struggle to relate to other health‐threatening situations and preventive measures they have not encountered before.

## 4 Discussion

The detailed results show that for the people with intellectual disability who participated in the study, the importance of health is expressed in feeling good, without symptoms of illness and being active. It is also related to the importance they place on a healthy lifestyle in terms of physical activity, healthy food, and avoiding health‐threatening situations. The participants define health in positive and negative terms. Health is associated with well‐being, following daily routines, and fulfilling the role of a learner. This positive portrayal of health is characteristic of modern trends, which capture health in a positive light and emphasise its social dimension (see: Taranowicz 2023).

Some of the participants define health from a negative perspective, as the situation of not experiencing illness, which is typical of negative definitions (see: Domaradzki 2013). The absence of illness is associated with not having unpleasant and painful discomfort in the body, which is indicative of well‐being. This aspect can be put as follows: if there are no symptoms that impede daily functioning, then one is healthy; if there are ailments that prevent activity, then one is sick.

Participants' perceptions of health issues do not include identification of diseases that do not produce noticeable and clear symptoms which would significantly impede their daily functioning. This brings with it the need for in‐depth knowledge of various aspects of health, which can be a challenge for people with intellectual disability. This group requires ongoing education about health in different phases of life, as well as support in accessing assistance and healthcare.

Health for people with intellectual disability means a certain kind of balance, which is expressed in activities performed in the family and social environment, as well as in the fulfilment of dreams. In a general sense, a healthy student usually attends school and experiences independence (Kuijken et al. 2016).

Health is associated with the need for independence and autonomy in social roles, which resonates more clearly in studies conducted among adults (Brolan et al. 2012; Cytowska and Zierkiewicz 2020). Survey results show low levels of autonomy in relation to health (see: Wullink et al. 2009). Adolescents internalise the messages of significant others and adopt them as an image of themselves, perceiving themselves as healthy or sick. Thus, some participants perceive their health through the lens of loved ones, especially the mother, who may form a symbiotic relationship with a child with a disability (Gumienny 2020). Studies show some reduction of autonomy and subjectivity in the context of health competence, as well as self‐perception according to the point of view of significant others. This can be explained by the fact that parents are one of the main sources of knowledge about health (Dam et al. 2022) and that they are involved in decisions about their children's health (Niedbalski 2023).

A healthy lifestyle relates to sports and physical activity. Adolescents motivate their need for physical activity in terms of taking care of their fitness, maintaining their body weight, losing weight, and oxygenating the body. Similar findings regarding physical activity can be found in studies by other authors (Caton et al. 2012; St. John et al. 2018). Despite knowledge about healthy lifestyles, physical activity is occasional and most often associated with physical education classes at school. Other studies also point to lower levels of physical activity among people with intellectual disability (Olsen et al. 2021; Scott and Havercamp 2018; Tyrer et al. 2020; Zwack et al. 2022).

Health is equated with healthy and unhealthy foods, which is consistent with the findings of Kuijken et al. (2016). Participants see the need to eat healthy foods and hydrate their bodies. The survey revealed the need to eat rationally, follow a diet, and limit excessive eating in order to maintain health. Unhealthy foods are associated with experiencing taste‐related pleasure, but these foods, according to participants' statements, do not predominate in their diets. Similar results were found in a study by Caton et al. (2012). According to the youngsters, it is also important to dress appropriately for the cold seasons, which is an important part of preventive health care (Bergstrom et al. 2014).

Participants were able to identify various health‐threatening situations, pay attention to land traffic accidents, and list stimulants and substances that harm health. The types of dangers listed indicate that people with intellectual disability distinguish a narrow range of dangers, as they rely mainly on individual experiences, without taking into account situations they have not encountered. Some failed to adequately recognise actual dangers. Few are aware that health can be threatened by experiencing physical and psychological aggression, or the abuse of modern technologies leading to impaired vision and addiction. Health, which is equated with a sense of security, can also be threatened by war, which triggers fear and anxiety.

Young people have knowledge of types of certain diseases and their symptoms. This includes the conditions most common in the general population. Participants are familiar with the basic patterns of medical advice and management during treatment, although this knowledge needs to be deepened. Some statements refer to a virus as the cause of illnesses, including the coronavirus that caused the Covid‐19 pandemic, which posed a threat to health and life, leading to the death of many people. The pandemic also affected the psychosocial functioning of people with developmental disorders (Lew‐Koralewicz 2022).

In medical emergencies, including injuries or burns, participants are aware of the elements of first aid related to protecting the wound from infection. In more serious cases, they recognise the need to call an ambulance. Pro‐health education is needed, including preventive education among people with intellectual disability that deepens their level of knowledge and skills in taking care of their health.

## 5 Conclusion

The main research finding is that the participants perceive health issues in an individual way and have varied knowledge on the subject.

The goal of the IPA was to explore the meanings that adolescents with intellectual disabilities attribute to health, their experiences, and how they perceive situations that threaten their health. This research offers valuable insights and contributes to the existing body of knowledge regarding health experiences and perceptions. Four key categories emerged from the participants' experiences: understanding health, perception of a healthy lifestyle, identification of health‐threatening situations, and experiences with illness.

The study highlights the diverse experiences of preferred lifestyles and offers insights into the perspectives and needs of adolescents with intellectual disabilities.

Given the age of the adolescents involved, there is a need to enhance their independence and autonomy in identifying symptoms of illness. It is also important to develop their ability to articulate their health and well‐being, which is crucial during medical consultations and the process of obtaining an accurate diagnosis and treatment. In light of these findings, we recommend implementing health interventions specifically designed for people with intellectual disabilities (McPherson et al. 2017).

We also recognise the importance of strengthening health education to enhance quality of life by promoting appropriate leisure activities, raising awareness, and developing practical skills for maintaining a healthy diet and staying fit. It is crucial to promote proper health and preventive behaviours, which play an important role in building the self‐determination of individuals with intellectual disabilities and supporting their decision‐making (Friedman et al. 2019).

The findings of this study can be integrated into educational and therapeutic programs, as well as in the person‐centred planning process and staff training (including teachers, therapists, personal assistants, social workers, volunteers, and others). Implementing health‐promoting measures tailored to the individual's needs will help prevent diseases and better address the unique needs and preferences of people with intellectual disabilities. These needs are also highlighted in other studies (Barrington et al. 2025; Danon et al. 2025; Wilson and Goodman 2011).

Future research could provide more in‐depth insights, particularly focusing on the needs related to health‐promoting decision‐making, physical activity, preventive health behaviours, and available support options. It is also essential to implement health‐promoting programmes specifically designed for individuals with intellectual disabilities who also experience coexisting disabilities and health conditions.

A limitation of the study is the challenges of analysing and interpreting the data as it comprises statements made by people with intellectual disability with varying communication and language skills. Young people were not always able to express their experiences in detail, requiring support from the researchers. Some participants spoke freely about their experiences, while others needed more time to reflect and needed additional time and assistance to understand the more complex issues (for example, clarification, elaboration). Participants responded at different paces, which, in some cases, prolonged the duration of the interview.

## Author Contributions

**Beata Gumienny:** conceptualisation, methodology, formal analysis, investigation, resources, writing – original draft, writing – review and editing, visualisation. **Aneta Lew‐Koralewicz:** conceptualisation, methodology, formal analysis, investigation, resources, writing – original draft, writing – review and editing, visualisation.

## Consent

Informed consent was obtained from parents and all participants in this study.

## Conflicts of Interest

The authors declare no conflicts of interest.

## Data Availability

The data that support the findings of this study are available on request from the corresponding author. The data are not publicly available due to privacy or ethical restrictions.
